# Expecting the Unexpected: Entropy and Multifractal Systems in Finance

**DOI:** 10.3390/e25111527

**Published:** 2023-11-09

**Authors:** Giuseppe Orlando, Marek Lampart

**Affiliations:** 1 Department of Mathematics, University of Bari, Via Edoardo Orabona 4, 70125 Bari, Italy; 2 Department of Mathematics, University of Jaen, Campus Las Lagunillas s/n, 23071 Jaén, Spain; 3 Department of Economics, HSE University, 3A Kantemirovskaya Ulitsa, St. Petersburg 190121, Russia; 4 IT4Innovations, VSB—Technical University of Ostrava, 17. Listopadu 2172/15, 708 00 Ostrava, Czech Republic; marek.lampart@vsb.cz; 5 Department of Applied Mathematics, VSB—Technical University of Ostrava, 17. Listopadu 2172/15, 708 00 Ostrava, Czech Republic

**Keywords:** entropy, multifractal analysis, financial time series, determinism, risk management, investments

## Abstract

Entropy serves as a measure of chaos in systems by representing the average rate of information loss about a phase point’s position on the attractor. When dealing with a multifractal system, a single exponent cannot fully describe its dynamics, necessitating a continuous spectrum of exponents, known as the singularity spectrum. From an investor’s point of view, a rise in entropy is a signal of abnormal and possibly negative returns. This means he has to expect the unexpected and prepare for it. To explore this, we analyse the New York Stock Exchange (NYSE) U.S. Index as well as its constituents. Through this examination, we assess their multifractal characteristics and identify market conditions (bearish/bullish markets) using entropy, an effective method for recognizing fluctuating fractal markets. Our findings challenge conventional beliefs by demonstrating that price declines lead to increased entropy, contrary to some studies in the literature that suggest that reduced entropy in market crises implies more determinism. Instead, we propose that bear markets are likely to exhibit higher entropy, indicating a greater chance of unexpected extreme events. Moreover, our study reveals a power-law behaviour and indicates the absence of variance.

## 1. Introduction

Entropy in finance and economics is a concept derived from thermodynamics and information theory, applied to study uncertainty and dynamics in financial and economic systems. As opposed to self-similarity, it quantifies disorder, randomness, and unpredictability in these systems, aiding in risk assessment, portfolio optimisation, and policy formulation [1]. Thus, entropy is a measure of the unexpected. For example, Jorion [2] attributes the abnormal losses during the 2008/9 financial crisis to the unexpected (the so-called unknown unknowns [3,4]). Frittelli [5] suggested a “minimal entropy martingale measure” based on the argument that, under some conditions, the said minimisation of entropy is equivalent to maximising the expected exponential utility of terminal wealth. Geman et al. [6] use entropy maximisation to represent the real-world ignorance of the “true” probability distributions. Kelbert [7] suggested weighted entropy for building optimal portfolios for risk-averse Kelly investments. In EEG analysis, entropy measures like approximate entropy (ApEn) and sample entropy (SampEn) assess brain activity complexity and randomness [8,9]. High entropy values indicate diverse brain activity, while low values suggest more regular patterns [10,11]. Entropy analysis helps researchers understand brain function, neurological conditions, and treatment effectiveness, contributing to advancements in neuroscience and diagnostic approaches [12].

With regard to the definition and use of entropy, Kolmogorov–Sinai entropy [13,14] is not well-suited for statistical applications. To address this limitation, ApEn [15] was introduced as a statistic for empirical and simulated data series. However, ApEn has drawbacks, leading to biased results that exaggerate regularity [16]. To overcome this bias, Richman and Moorman [17] (see also [18]) proposed an alternative statistic known as SampEn. When considering the input parameters *m* (sub-series length), *r* (tolerance/similarity criterion), and *N* (data length), the authors of [19] found that ApEn and SampEn are highly sensitive to the choice of parameters, particularly for very short data sets with N≤200. For this reason, they recommended using *N* larger than 200, setting *m* to 2. Similarly, the authors of [20] reported a significant dependency of both ApEn and SampEn on input parameters. Specifically, when *m* was chosen, both ApEn and SampEn tended to decrease as *r* increased, except for low values of *r* combined with high values of *m*. The decreasing trend showed steeper slopes for lower values of *m*. Similarly, for a chosen *r*, both ApEn and SampEn tended to decrease as *m* increased. In essence, the analysed time series exhibited more regularity (lower entropy values) for higher similarity tolerances and longer subseries lengths. In their study, Zhang et al. [21] compared ApEn, SampEn, and Permutation Entropy (PE) and found that SampEn is the least influenced by signal length and remains unaffected by the signal’s amplitude and phase. The variations in the three entropy measures are solely related to frequency.

Olbrys et al. [22] investigated changes in sequential regularity in European and U.S. stock market indices during the Global Financial Crisis and COVID-19 pandemic and found increased regularity in stock market returns during both turbulent periods. However, as demonstrated by the authors of [23], both ApEn and SampEn are very sensitive to the presence of spikes. For narrowband line spectra test signals, the presence of spikes leads to an increase in both ApEn and SampEn. For test signals that are better modeled as broadband random processes, the presence of spikes results in a decrease in entropy. In the case of real RR records, where spikes are often caused by QRS detection errors [24], the presence of spikes also leads to a decrease in entropy (for details on RR and QRS see Appendix A). Thus, lower entropy reported by some studies in the literature [22,25,26] may not be due to “increased regularity in stock market returns", but rather because of increased volatility. Similarly, Wang et al. [27] found that the market efficiency (i.e., multiscale entropy) of the S&P 500 Index, gold, Bitcoin, and the US Dollar Indexe during the extreme event of the COVID-19 pandemic, at all scales, decreased sharply and persistently during February–March 2020.

Regarding fractality, Mandelbrot boldly claimed that fractal tools are likely to retain their significance in finance because of the inherent roughness in price data, and fractal geometry serves as the most appropriate framework for understanding this concept of roughness in both natural and financial contexts [28]. Fractal structures have implications for market efficiency, challenging the notion that financial markets can be unequivocally categorised as entirely efficient, as suggested by the Efficient Market Hypothesis (EMH). Instead, it suggests a more nuanced perspective, wherein financial markets exhibit a mixture of both efficiency and inefficiency. At times, markets provide fair returns on investments, while at other junctures, investors might experience unexpectedly substantial profits or significant losses [29]. Fractal analysis, relying on metrics such as the Hurst exponent and entropy-based indicators has been also used for forecasting [30]. Fractality is associated with phenomena characterised by long-range memory and non-locality. These phenomena have been studied using fractional calculus, which extends the concepts of differentiation and integration to real or complex orders [31,32]. Mandelbrot et al. [33] suggested fractional Brownian motions [34,35] as applicable to natural phenomena and finance. Machado et al. [36] empirically confirmed a strong correlation between the entropy and the value of the fractional order.

Natural processes, such as population dynamics and neurosystems, often exhibit complex behaviours characterised by intermittent bursts of apparent randomness [37,38,39,40]. These behaviours encompass both long-term and short-term dynamics and have found applications in finance and economics [41,42].

Evidence of strong deterministic elements concealed within these stochastic features have been found in real-world data [43]. Surprisingly, the deterministic approach yielded results similar to the stochastic one [41,44], revealing the full dynamical systems landscape and offering potential control mechanisms [45,46], challenging conventional views and opening new avenues for understanding and influencing these processes [47,48,49].

Our research aims to determine whether seemingly stochastic financial data might exhibit underlying determinism and anticipate statistically abnormal losses. On the matter of discerning between the output of a stochastic process and that of a deterministic process, Radunskaya [50] puts forth an impossibility theorem. This theorem asserts that a time series generated by deterministic B-processes (essentially a process isomorphic to a Bernoulli process is observationally equivalent to the output of a continuous-time Markov process with a finite number of states). As a result, randomness must be validated through alternative methods such as calculating the correlation dimension, Lyapunov exponents, entropy, Hurst exponent, and so on. To investigate this, we employ fractal analysis and entropy. If such determinism exists, we also explore whether entropy correlates with market conditions, specifically when a market downturn is positively associated with changes in entropy. To attain our objectives, we suggest an innovative approach that involves data segmentation and detrending through differencing. This process reduces the influence of underlying trends and enhances the accuracy of change point identification. Within each segment, we calculate key metrics such as SampEn, mean, and volatility (i.e., standard deviation). Then, Spearman rank correlation, which quantifies the relationships between entropy and the aforementioned statistics, is computed. The proposed methodology not only enhances data segmentation but also rectifies a common error related to SampEn. Unlike ApEn, which remains unaffected by changes in mean and volatility, SampEn decreases as volatility rises. Consequently, certain studies may report reduced entropy during market crises, whereas it actually signifies heightened volatility. Our findings challenge some of the existing literature [22,25,26,51], highlighting the potential ambiguity between volatility changes and bear markets. The latter does not necessarily imply increased determinism; instead, turmoil is more likely to align with heightened entropy, indicating a greater likelihood of extreme events [52]. Finally, our study reveals a power-law behaviour and suggests the absence of variance, aligning with earlier research by Grobys [53]. This prior work offers a compelling explanation for the high rate of scientific replication failure in many studies [54], pointing to the use of inappropriate research methodologies within specific environments, especially in the field of financial economics [55,56]. Together with Grobys, we question the existence of Lévy process [57,58], demonstrating that the power-law exponent for the returns of our specific equity index is significantly less than 3.

The rest of this paper is arranged as follows. Section 2 first describes the data and provides references, while the second part explains the methodology for data partitioning and relevant computations. An explanation, through simulations, of the relationship between entropy and shocks in mean and volatility is also provided. Section 3 begins with an analysis of the data in terms of stochasticity through spectral analysis. It then examines evidence of determinism and self-similarity with fractal analysis, and finally, conducts a deep dive into self-similarity by means of entropy. The last section presents the conclusion.

## 2. Materials and Methods

### 2.1. Data

For the analysis, we have considered both real data and a simulated Brownian motion for comparison (for a connection between macroeconomic business cycles, determinism, and financial stochastic Ornstein–Uhlenbeck process, see Orlando et al. [59]). The former refers to USA equities, and the latter is a signal commonly used in finance to model a stochastic process $y_{t}$ such as the Ornstein–Uhlenbeck process [60]

$$dy_{t}=-\varphi \;y_{t}\;dt+\sigma \;dB_{t}$$

where φ>0 and σ>0 are some parameters and $B_{t}$ denotes a standard Brownian motion [61,62,63,64].

#### 2.1.1. Real Data

**Figure 1:**
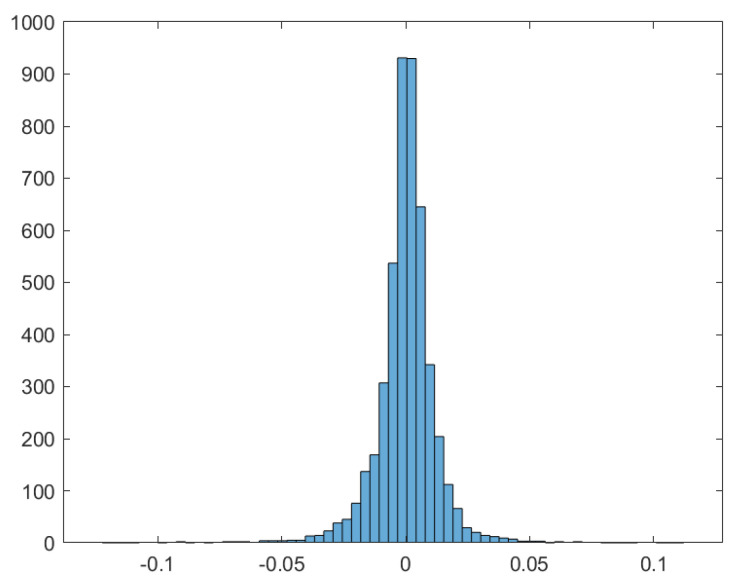
NYSE: Histogram of daily log-returns.

The data consist of daily New York Stock Exchange (NYSE) U.S. Index values from 12 January 2004 to 26 September 2023. Table 1 and Figure 1 present summary statistics of daily log-returns and their corresponding histogram, respectively. It is evident that these data exhibit negative skewness and positive kurtosis, which are common characteristics in financial data [52,65].

In addition, for detailed entropy analysis, daily data of 1136 constituents of the NYSE have been analysed from [66] since its inception to 12 December 2022. A Matlab routine was created to read individual CSV files containing daily stock quotes for each NYSE stock and perform the analysis. Empty daily value records were automatically filtered out. Furthermore, if the data records for a specific stock consisted of fewer than 40 data points, we did not include that security in our analysis. As a result, out of the initial 1145 securities, we excluded 9 leading to 1145 − 9 = 1136 securities.

#### 2.1.2. Simulated Data

A coloured noise signal is a function with a power spectral density (PSD) that follows a power law of the form

$$S\left(f\right)=\frac{L(f)}{{\left|f\right|}^{\gamma }}$$

where γ (i.e., the inverse frequency power) is a real number in the interval [−2,2] and L(f) is a positive, slowly-varying or constant function [67].

When γ=0, the signal is white noise, with L(f) being a constant directly proportional to the variance of the process. On the other hand, when γ=2, the signal is a brown noise nonstationary process with stationary increments (i.e., a Brownian motion). Brown noise is the integral of white noise [67] and it is extensively used for modelling financial processes [35,63,68,69].

### 2.2. Sample Entropy

As mentioned in the introduction, to gauge the level of regularity in a time series, approximate entropy (*ApEn*) was introduced [15]. However, *ApEn* has some drawbacks [16] that led to the development of the sample entropy (*SampEn*). *SampEn* provides an estimate of the conditional probability [16,18,70]. Denoted *m* as the embedded dimension, *SampEn* evaluates the likelihood that two comparable sequences, consisting of *m* consecutive data points, will remain similar even when additional consecutive data points are introduced to the sequences. Thus, *SampEn* quantifies complexity primarily at a specific time scale through a sampling approach using the formula

(1) $$SampEn\left(m,r\right)=\lim_{N\to \infty }-\ln\frac{P^{m}\left(r\right)}{Q^{m}\left(r\right)}$$

where $P^{m}\left(r\right)$ represents the probability that two sequences will match for *m* + 1 points and $Q^{m}\left(r\right)$ denotes the probability that two sequences will match for *m* points, with a tolerance of *r*, while excluding self-matches.

Parameters

Used parameters throughout the paper are: dim = 1 (embedded dimension); r = 0.2 std (tolerance); tau = 1 (delay time for downsampling); metric = Chebychev of utilised MATLAB [71] functions [72,73]; for a sensitivity analysis see Appendix B.

### 2.3. Spectral Analysis

Power spectrum and spectral analysis find application in economics and finance, particularly when examining data characteristics, especially during periods of economic expansion and recession. For instance, Strohsal et al. [74] employ spectrum estimation to assess the properties of financial cycles and describe their interactions in the frequency domain. An advantage of this analysis lies in its ability to unveil relationships at frequencies that might remain concealed in the time domain. Other applications are explored by DiMatteo et al. [75], who investigate scaling structures, and by Benedetto et al. [76], who apply spectral analysis to volatility indices, oil, and natural gas prices to reveal information flows between variables.

Spectral analysis involves the estimation of a signal’s power spectrum (PS) based on its time-domain representation. Spectral density describes the frequency components of a signal or stochastic process [77]. Essentially, the spectrum dissects the signal or process into its constituent frequencies, unveiling any periodic patterns, thereby enabling the assessment of local signal regularity. This capability to characterise signal regularity is of utmost importance, especially when dealing with phenomena that lack a distinct scale. Signals exhibiting scale-free dynamics, as commonly observed in various fields like geophysics, biomedical signal processing, internet traffic, finance, etc., serve as examples of such phenomena, as highlighted in [48]. When applying analysis techniques to data, inherent assumptions come into play. For instance, when using autocorrelation or power spectral density (PSD) estimation, there is a presupposition that the data is translation invariant. This implies that essential signal statistics like mean and variance remain constant over time. In contrast, signals devoid of a characteristic scale exhibit scale invariance, where signal statistics remain unaltered regardless of stretching or compressing the time axis. Traditional signal processing methods often prove inadequate in describing these signals or distinguishing between signals with varying scaling behaviours.

Spectral analysis encompasses both nonparametric and parametric methods [77,78]. Nonparametric methods involve segmenting time-domain data, applying Fourier transforms to each segment, computing the squared magnitude of the transform, and then summing and averaging the results. Variations of this approach include nonparametric methods such as the modified periodogram, Welch, Bartlett, and the Blackman–Tukey methods. These methods are data-driven and do not necessitate prior knowledge of the data or a predefined model. In contrast, parametric methods are model-based approaches. These methods construct a signal generation model using a set of parameters that can be determined from the observed data. Subsequently, the algorithm derives the power spectrum implied by the model, relying on the constructed model and the estimated parameters.

As the periodogram, by itself, is not a reliable estimator of the true power spectral density for a wide-sense stationary process, we have employed Welch’s method to decrease the variance of the periodogram. This is achieved by segmenting the time series, typically with some degree of overlap, as described in [78]. In Welch’s approach, an adjusted periodogram is computed for each segment, and these estimates are then averaged to generate the overall estimate of the power spectral density. Given the wide-sense stationarity of the process and the use of PSD estimates from different segments, the modified periodograms provide nearly uncorrelated estimations of the true PSD. The act of averaging these estimates effectively diminishes variability [79]. Typically, these segments are multiplied by a window function, like the Hamming window. As a result, Welch’s approach essentially entails averaging these adjusted periodograms. The introduction of overlap in the segments ensures that data values at the edges of one segment, tapered by the window, extend beyond the edges of adjacent segments. This approach serves to prevent the loss of information that can result from windowing, as discussed in [79].

### 2.4. Multifractality and Determinism

As explained by Mandelbrot [28,80], fractal geometry provides an intrinsic measure of roughness, marking the inception of a quantitative theory dedicated to roughness in all its forms. Traditional definitions of steepness involve derivatives of height along inclines, but fractal geometry introduces a unique approach. In fractal/multifractal models of price variation, the ratio of logarithmic height increments to logarithmic distance increments has a consistent limit, denoted as α=1/2. This property is both an advantage, given its uniformity, and a limitation, as it cannot be adjusted as a fitting parameter in financial data analysis. Conversely, fractal/multifractal models offer the flexibility of α≠1/2.

Arguments against the Brownian model were based on the assertion that α=1/2 represents mild roughness, while the roughness in financial data is more extreme, excluding α=1/2. Various alternative models emerged to address these concerns by introducing “fixes” to avoid anomalies like discontinuity and divergent moments. One common feature among these fixes is the automatic resetting of local roughness to 1/2 [28]. One such fix is stochastic volatility, which assumes that short records follow Brownian motion, but the variance in this motion changes periodically. However, this approach shifts the modeling burden to the process governing volatility variations. Additionally, if volatility changes slowly, it implies α=1/2, which contradicts the roughness observed in financial data [28]. In summary, the argument against the Brownian model in finance led to the development of alternative models with fixes, but these fixes often implicitly assume a local roughness of α=1/2, which may not align with the empirical evidence [28].

As previously discussed in the introduction, real-world data has provided indications of multifractality and determinism. To elaborate, Orlando et al. [44] conducted an extensive examination across various financial instruments and indices. This encompassed a wide spectrum, including the Financial Stress Index, swaps, equities, and both emerging and developed markets, as well as corporate and government fixed income securities, spanning short and long maturity bonds. Stoop et al. [43] employed a low-dimensional deterministic approach on traditionally stochastic data, uncovering previously unnoticed deterministic patterns. Stochasticity prevailed on short timescales, while determinism emerged on intermediate timescales. This observation provides insights into the underlying structures behind seemingly random events and, furthermore, revealed similarities in market behaviour, global indicators, and neuronal firing patterns [37], underscoring the theoretical significance of studying this system category. Indeed, Orlando and Bufalo [41] identified the same pattern in the realm of credit. They introduced a deterministic approach for modeling time series, including indices like Moody’s Seasoned Aaa Corporate Bond Yield Relative to Yield on 10-Year Treasury Constant Maturity and the ICE BofA U.S. High Yield Index Option-Adjusted Spread. Their research concluded that a neural deterministic model effectively captures the dynamics of credit bubbles and bursts, as well as patterns of both low and high volatility, autoregression, cointegration, and heteroscedastic volatility.

### 2.5. Segmentation and Correlation

Rather than analysing the time series in its original form, the initial step involves removing the trend through differencing. Subsequently, the data is segmented following the methodology outlined by Lavielle [81], Lavielle et al. [82], Killick et al. [83] and implemented in [84].

Let $I_{k}$ represent the periodogram of the sequence ${\left\{Y_{j}\right\}}_{j=1}^{N}$ calculated within segment *k* at the frequency ω. Suppose that the energy of the process within specific frequency intervals $\left[\lambda_{j},\xi_{j}\right)$, where 1≤j≤J, in the range of [0,π], undergoes a sudden and unknown change at some point. The energy of $\left(Y_{\theta_{k-1}+1},\ldots Y_{\theta_{k}}\right)$ in the frequency band $\left[\lambda_{j},\xi_{j}\right)$ can be written as

$$F_{kj}=\int_{\lambda_{j}}^{\xi_{j}}I_{k}\left(\omega \right)d\left(\omega \right)\;.$$

The suggested [81,82] contrast function for detecting the changes is

$$J_{n}\left(\theta ,y\right)=-\frac{1}{n}\sum_{k=1}^{K^{*}}\left(n_{k}\sum_{j=1}^{J}F_{kj}^{2}\right)\;$$

where $K^{*}$ denotes the unknown number of segments (resp. change points).

The detrending enhances the ability to identify change points more effectively. In each segment, SampEn, mean, and volatility (standard deviation) are computed on the original time series. Subsequently, the Spearman rank correlation is calculated between SampEn and both the mean and volatility. This methodology is new, and in addition to the aforementioned advantage of improved segmentation, it circumvents a common mistake associated with the application of SampEn. In fact, it can be demonstrated that while ApEn remains unaffected by changes in mean and volatility, SampEn decreases as volatility increases. Consequently, some research may inadvertently report a decrease in entropy during market crises [22,25,26], when it is actually the result of heightened volatility.

**Figure 2:**
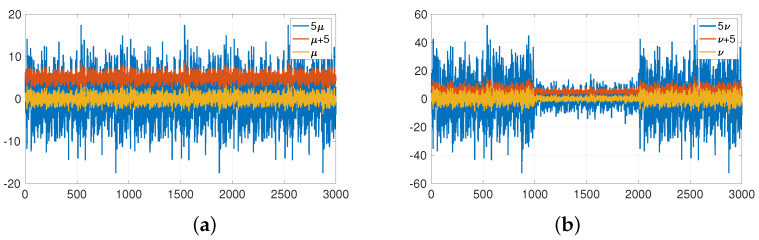
Time histories of: (**a**) μ, (**b**) ν and their mean and volatility shifts.

To demonstrate the statement above, we generated a random time series from a Gaussian-normalised distribution and shifted the mean and volatility. The result shows that the ApEn does not change in any case, while the SampEn changes in response to volatility changes. To show this observation, a generic example can be constructed. A random Gaussian standardised vector of length 1000 copied 3 times, denoted μ, has the SampEn of 2.1172. On the other hand, vector ν composed of three scaled copies of the same random Gaussian standardised vector, that is multiplied by 3 for the first 1000 values, multiplied by 1 for the second 1000 values, and by 3 for the third 1000 values, has the SampEn of 0.6429. Corresponding graphs of μ and ν and their mean and volatility shifts are shown in Figure 2a,b, respectively. All corresponding ApEn and SampEn are summarised in Table 2.

Similarly, if the same random Gaussian standardised vector is multiplied by 1 for the first 1000 values, by 3 for the second 1000 values and by 1 for the third 1000 values, it has the SampEn of 1.1337. Therefore, once again, the reduction in entropy (from 2.0950 to 1.1337) only reflects a change in volatility and not in determinism.

## 3. Results and Analysis

In this section, we analyse if the considered NYSE index looks stochastic (e.g., a Brownian motion), displays dominant frequencies, and if there is evidence of self-similarity in terms of fractality. Finally, a deep dive into self-similarity is conducted by means of entropy. Segmentation was conducted following the approach outlined in Section 2.5, employing the *findchangepts* Matlab function with the “std” metric for detecting changes in standard deviation. Additionally, the parameter *MinThreshold* was set to 50. For more comprehensive information, refer to [84].

### 3.1. Power Spectral Density

In the following, our aim is to discern whether the NYSE displays characteristics akin to a stochastic process through power spectrum and spectral examination. This scrutiny seeks to unveil whether determinism lies hidden beneath what might appear as mere “stochastic activity” [43]. Should such concealment become apparent, it would necessitate an alternative approach, like fractality and entropy assessment.

**Figure 3:**
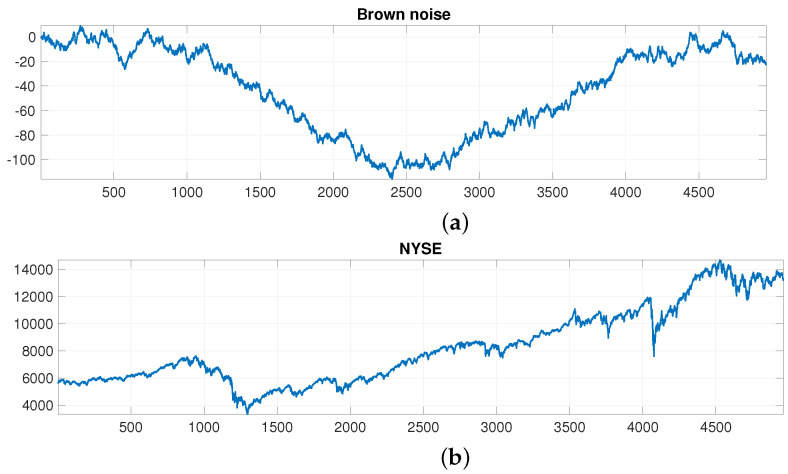
Time series of: (**a**) Brown noise and (**b**) NYSE.

**Figure 4:**
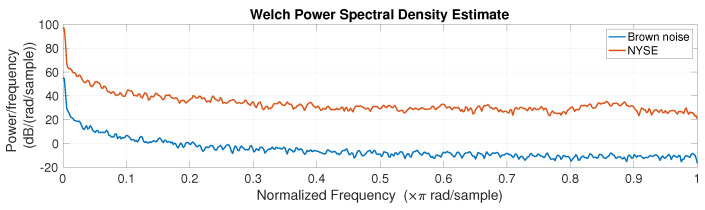
Welch Power Spectral Density Estimate for Brownian noise (blue) and the NYSE (red).

Let us start by analysing whether the NYSE index has some regularity or prevalent frequency. For better understanding, it can be useful to compare the NYSE with Brownian noise, also known as brown noise or red noise, i.e., as already mentioned, a type of noise produced by Brownian motion often used in finance for modeling erratic motion [34,35,60]. Figure 3a displays a randomly generated brown noise next to the plot of the NYSE, Figure 3b. Apparently, the two time series, except for causal changes, look similar. To confirm this, Figure 4 compares the power spectral density (PSD) estimates of the two analysed time series, thereby verifying their similarity.

### 3.2. Power Law Process Estimation

**Figure 5:**
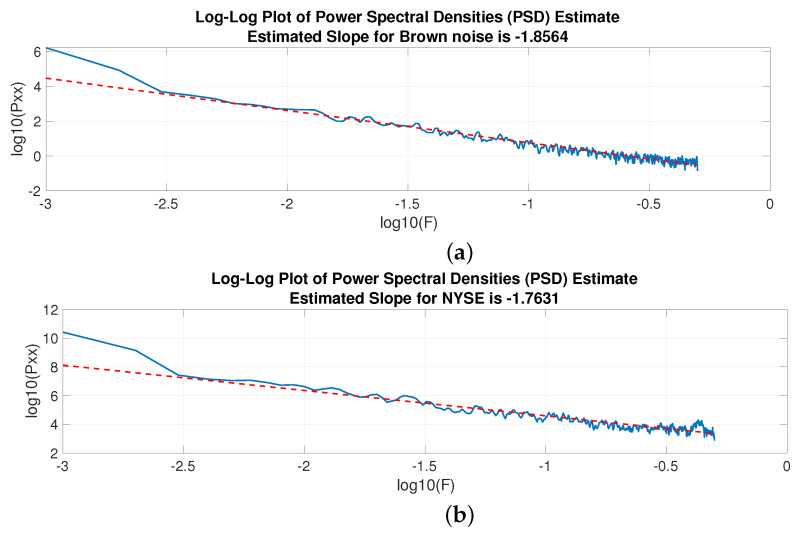
Log-log plot of the PSD of brown noise (**a**) and the NYSE (**b**). Interpolating line (red), log-log of the PSD (blue).

Signals exhibiting scale-free dynamics often display autocorrelation or power spectral densities (PSD) that adhere to a power-law pattern. This power-law process can be characterised by a PSD with the following form:

$$C=abs{\left(\omega \right)}^{-\beta }$$

where *C* represents a positive constant and β denotes the exponent. In the context of brown noise, the theoretical exponent is fixed at 2. One approach to determining the exponent of a power-law process involves fitting a least-squares linear regression to a logarithmically transformed plot of the PSD. Figure 5a,b show the estimate for the two considered time series.

As discussed in the work of London et al. [85], the value of the exponent in the power law has implications for the scaling (decaying) behaviour of the tails in the empirical probability density function (PDF). For example, Bouchaud et al. [86] describe a power law behaviour, expressed as $p\left(x\right)∼x^{-\beta }$. For example, take the Pareto wealth distribution P(W), a classic economic power law depending on individual wealth *W*. In its asymptotic tail, this distribution is typically expressed as a power law:

$$P\left(W\right)∼\frac{W_{0}^{\mu }}{W^{1+\mu }}\;\mathrm{for}\;W\gg W_{0}\;.$$

Within this equation, the parameter μ determines how the distribution behaves for large values of *W*. A smaller μ indicates a slower decay and a more significant disparity between the wealthiest and the least affluent individuals. In the context of financial markets, as shown by Gopikrishnan et al. [87] and Bouchaud et al. [86], it is worth noting that the distribution of price increments can be effectively modeled as a power law with μ typically around 3. This value of μ falls well outside the stable Lévy regime of 0<μ<2. Other studies suggest that the tails decay in a manner akin to the log-normal distribution (as originally proposed by Bachelier in 1900 and referenced in Cont et al. [88]). This issue is closely related to whether the second moment of the empirical distribution is finite. In general, for continuous distributions with tails that decay more rapidly than $1/x^{3}$, the process’s second moment exists, as noted by Bouchaud et al. [89] and Stuart et al. [90].

Our findings, indicating a power law behaviour and a decay lower than $1/x^{3}$, are consistent with recent research conducted by Grobys [53]. Grobys analyzed time series data for the S&P 500, gold, crude oil, the USD to GBP exchange rate, and Bitcoin. Grobys’ study revealed that the daily variances of all five major asset markets follow power law processes. Significantly, these results maintain consistency across various data samples and analytical methods. Furthermore, the study strongly suggests that there is no statistical evidence of the variance in any of the analysed asset markets. For additional insights into scaling spectral density, the behavior of power spectra, and the application of the Hurst exponent in finance, see Di Matteo [75].

### 3.3. Multifractal Analysis

**Figure 6:**
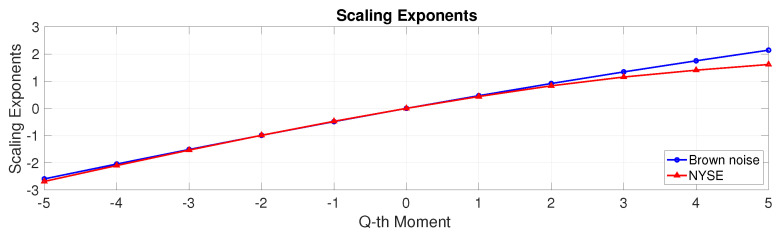
Multifractal analysis. The scaling exponents for the brown noise process are a linear function of the moments, while the exponents for the NYSE show a departure from linearity.

Fractal analysis can unveil the underlying structural intricacies of a signal that may elude traditional signal processing techniques. Multifractal analysis involves the investigation of whether there is a presence of power-law scaling for diverse statistical moments across different scales. If this scaling behaviour is defined by a single scaling exponent, or equivalently, if it manifests as a linear function of the moments, then the process is classified as monofractal. If the scaling behaviour (with respect to scale) is a non-linear function of the moments, the process is categorised as multifractal (see Figure 6).

### 3.4. Multifractal Spectrum

**Figure 7:**
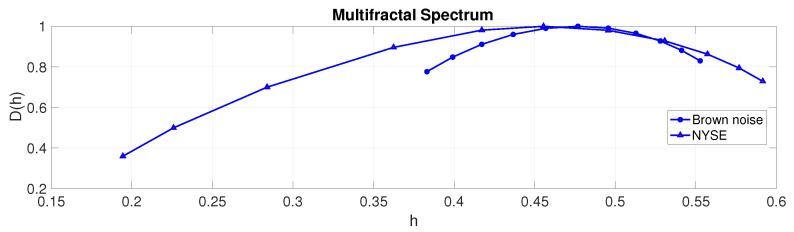
Multifractal spectrum. Brown noise appears to be a monofractal signal characterised by a cluster of scaling exponents around 0.48 and a support between [0.38 0.55]. The NYSE appears to be a multifractal signal characterised by a much wider range of scaling exponents between [0.19 0.59] around its peak of 0.46.

The multifractal spectrum illustrates the array of scaling exponents for a signal. Similarly, it serves as a gauge of the temporal variations in local regularity within a signal. In the case of a monofractal signal, uniform regularity persists throughout time, resulting in a multifractal spectrum with limited breadth. Conversely, a multifractal signal displays fluctuations in signal regularity over time, yielding a multifractal spectrum with broader support. As depicted in Figure 7, brown noise exemplifies a monofractal process, while the NYSE index displays multifractal characteristics, consistent with previous research findings [91,92,93].

### 3.5. Entropy

As previously noted, the examination of self-similarity can be approached through various angles, including fractality. However, while the latter aids in grasping the essence of time series, it offers limited insights into shifts in determinism linked to market conditions. Hence, the central research inquiry revolves around the persistence of self-similarity, prompting our recourse to entropy analysis to investigate this matter. As in the previous section, we first compare the brown noise versus the NYSE. The SampEn of the first difference is 5.1445, versus 5.9674 for the NYSE.

A bullish market is characterised by a positive average return on investment, whereas a bearish market is the opposite. Let us start by examining the detrended NYSE index. As mentioned previously, our research question revolves around whether self-similarity and entropy change when the market undergoes a shift. To explore the potential relationship between entropy and bullish/bearish markets, we partitioned the NYSE data using the methodology outlined in [81,82,83].

**Figure 8:**
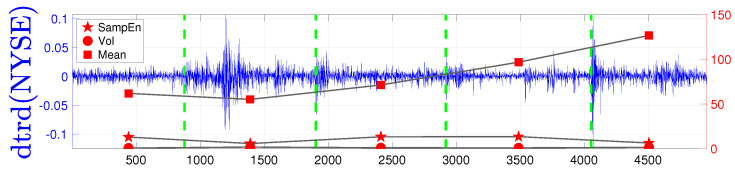
Time series of **daily** detrended NYSE data and output of SampEn, standard deviation, and mean computed on sub-intervals with endpoints as change points. For SampEn, embedded dimension was 1 and *r* was set as 20% of the standard deviation of tested data. In this case corr(SampEn(NYSE),Mean(NYSE))=0.4 where Spearman’s Rho type correlation was utilised. Notably, entropy increases when there is a negative change in the mean, while changes in volatility do not appear to have a significant impact on entropy.

**Figure 9:**
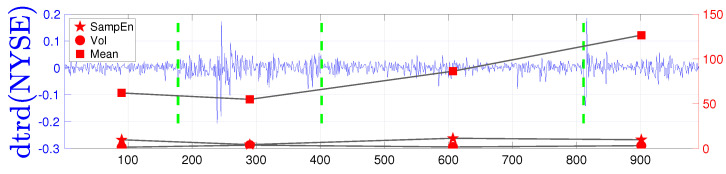
Time series data from the detrended **weekly** downsampled NYSE data and the results of SampEn, standard deviation, and mean calculations performed on sub-intervals defined by change points. For SampEn, embedded dimension was 1 and *r* was set as 20% of the standard deviation of tested data. In this case corr(SampEn(NYSE),Mean(NYSE))=0.8 where Spearman’s Rho rank correlation was utilised. Notably, entropy increases when there is a negative change in mean, while changes in volatility do not appear to have a significant impact on entropy.

**Figure 10:**
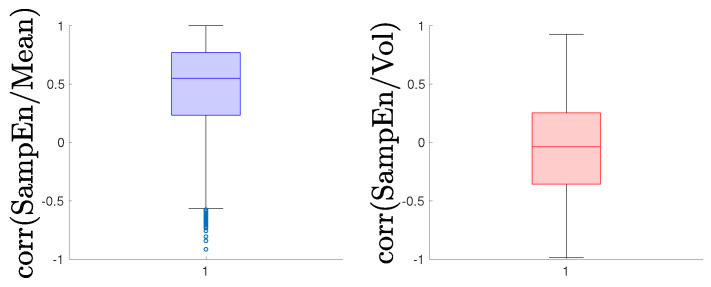
Spearman rank correlation between *SampEn* and mean (blue), and *SampEn* and volatility (red), calculated over 1136 constituents of the NYSE. Note the negative outliers in the mean. As shown, there is a positive correlation between entropy and changes in the mean, whereas this is not the case for changes in volatility.

For each partition, we computed the mean, standard deviation, and SampEn. Contrary to some findings in the literature [22], we observed that, in the analysed data, self-similarity is not dependent on volatility. Instead, there is evidence of a positive correlation between entropy and the mean. Particularly during a downward (bearish) market, entropy increases. This trend is consistent at different frequencies, such as daily (see Figure 8), as well as on a monthly basis (see Figure 9). This finding contrasts with some prior literature [22,25,26,51] and, as mentioned, is due to possible confusion between changes in volatility and a bear market, where the latter does not signal an increase in determinism. On the contrary, a turmoil should increase the likelihood of an extreme event and thus, should be correlated with an increase in entropy. Drawing a parallel with ECG entropy recorded in healthy individuals during wakefulness compared to deep sleep [11], this may suggest that bullish and bearish markets have a similar effect on entropy. Indeed, Figure 10 shows negative outliers in the mean and a positive correlation between entropy and changes in the mean, while this is not the case for changes in volatility. Furthermore, Table 3 summarises the results for the constituents of the NYSE index. It reveals evidence of a correlation between the mean (representative of market conditions) and SampEn, with a value of 0.4510. Additionally, the presence of outliers is evident, as indicated by the skewness, while the excess kurtosis stands at 0.5456 (compared to the Gaussian distribution’s value of 0). Lastly, the correlation coefficient is 0.7333, affirming the strong relationship between entropy and market conditions. This might be a consequence of the characteristics of the distributions of the mean (which displays few very negative outliers) versus the distribution of the volatility (which displays a more evenly distributed number of outliers).

## 4. Conclusions

Our research aims to uncover hidden determinism within ostensibly stochastic financial data and investigate its relationship with market conditions, particularly during market downturns. Entropy serves as a valuable measure for assessing the degree of complexity within systems by quantifying the average rate of information loss regarding a phase point’s position on the attractor. When dealing with multifractal systems, a single exponent is insufficient to capture their dynamics. Instead, a continuous spectrum of exponents is required. In our investigation of the New York Stock Exchange (NYSE) index and its constituent components, we have uncovered multifractal characteristics. Furthermore, we have demonstrated the effectiveness of using entropy as a tool to identify market conditions, whether they are bearish or bullish. This approach provides valuable insights into quantification of the unexpected or the so-called unknown unknowns [3,4]. Specifically, we have proposed a methodology that involves detrending the data through differencing, thereby improving the precision of change point identification for segmentation. Within each data segment, we have computed key metrics, including SampEn, mean, and volatility, and have quantified the relationships between entropy and these statistics using Spearman rank correlation.

In terms of entropy, our findings challenge conventional wisdom, as we have demonstrated that SampEn increases with price downfall. This revelation rectifies a common misconception wherein reduced entropy during market crises might be inadvertently interpreted as increased self-similarity or determinism. Our research highlights the potential confusion between shifts in volatility and bear markets. Once the trend has been removed, one may clearly see that. Importantly, our study suggests that bear markets do not necessarily indicate increased determinism. Instead, turmoil is more likely to correlate with heightened entropy, signifying a greater likelihood of extreme negative returns and loss bearing outliers [52].

In terms of power law behaviour and the absence of variance of variance, our results are consistent with previous literature by Grobys [53], who critiqued the Lévy process [57,58]. As outlined by Grobys, the issues can be attributed to the specific nature of the data and the statistical methods used, contributing to replication failures in top-tier financial (and other types of) journals [55,56]. Drawing a parallel with ecology, Pyke challenged the Lévy walk model [94,95] for its lack of realism as it neglects directional aspects in individual movements and demonstrates lower foraging efficiency compared to more realistic models. Additionally, the evidence supporting the occurrence of Lévy walks in actual organisms is limited, despite some claims [96]. Similarly, Levernier et al. [97] demonstrated that the Lévy hypothesis, suggesting that inverse square Lévy walks are optimal search strategies leading to efficient encounters with sparse, randomly distributed targets (which is applied to explain various biological behaviours), is unfounded.

## Figures and Tables

**Table 1:** NYSE: Summary statistics of daily log-returns.

Mean	Minimum	Maximum	Std. Dev.	Skewness	Kurtosis
0.00016	−0.1259	0.1152	0.0125	−0.6500	16.4272

**Table 2:** Values of the ApEn and SampEn for the generic example of Gaussian normalised distributions μ and ν and their shifts in mean and volatility.

	Original data μ	Mean shift of μ	Volatility shift of μ
ApEn	2.2217	2.2217	2.2217
SampEn	2.1172	2.1172	0.6429
	Original data ν	Mean shift of ν	Volatility shift of ν
ApEn	2.1632	2.1632	2.1632
SampEn	1.0129	1.0129	0.0811

**Table 3:** Correlation between *SampEn* and mean/volatility.

	Correlation	
	SampEn/Mean	SampEn/Vol.
Mean	0.4510	−0.0509
Mode	0.7333	0.1152
Ex. Kurtosis	0.5456	−0.7753
Skewness	−1.0548	−0.0626

Spearman rank correlation between *SampEn* and mean, and *SampEn* and volatility, calculated on each partition. The average number of partitions is 9.9287 and the mode is 10.

## Data Availability

The data presented in this study are available on request from the corresponding author.

## Appendix A. RR Interval and QRS Complex

The RR interval represents the time duration between successive heartbeats in an electrocardiogram (ECG) or electrocardiograph [98]. It is used to calculate the heart rate, also known as the rhythm rate, which indicates the number of heartbeats per minute. The QRS complex in an electrocardiogram (ECG) represents the combined graphical deflections corresponding to the depolarization and contraction of the right and left ventricles of the heart [98]. It is the most prominent part of the ECG tracing and typically lasts 80 to 100 ms in adults (shorter in children). The QRS complex consists of the Q, R, and S waves, occurring rapidly and reflecting a single event. The Q wave is a downward deflection following the P wave, followed by the upward deflection of the R wave, and then a downward deflection of the S wave. The T wave appears after the S wave, and in some cases, an additional U wave may follow the T wave.

## Appendix B. Entropy Sensitivity Analysis

In terms of sensitivity to different choices of embedding and tolerance, Table A1 and Table A2 show that SampEn exhibits greater stability than ApEn. Specifically, for SampEn, the most consistent results are obtained with an embedding value of 1. It is worth noting that for extremely low tolerance levels (i.e., *r*), the results are understandably not significant.

**Table A1:** ApEn and SampEn of detrended NYSE with respect to the embedded dimension and tolerance.

Embedded Dimension	Tolerance	ApEn	SampEn
1	0.4 std(dtrd(NYSE))	1.3739	5.4112
2	0.4 std(dtrd(NYSE))	1.2504	5.4112
3	0.4 std(dtrd(NYSE))	1.0909	16.323
1	0.3 std(dtrd(NYSE))	1.6410	5.6300
2	0.3 std(dtrd(NYSE))	1.4751	16.324
3	0.3 std(dtrd(NYSE))	1.2171	NaN
1	0.2 std(dtrd(NYSE))	2.0146	5.9674
2	0.2 std(dtrd(NYSE))	1.7407	16.324
3	0.2 std(dtrd(NYSE))	1.2514	NaN
1	0.02 std(dtrd(NYSE))	3.3943	16.324
2	0.02 std(dtrd(NYSE))	0.7004	NaN
3	0.02 std(dtrd(NYSE))	0.0208	NaN
1	0.002 std(dtrd(NYSE))	1.9683	16.324
2	0.002 std(dtrd(NYSE))	0.0108	NaN
3	0.002 std(dtrd(NYSE))	−0.0002	NaN

**Table A2:** ApEn and SampEn of detrended Brownian noise (BN) with respect to the embedded dimension and tolerance.

Embedded Dimension	Tolerance	ApEn	SampEn
1	0.4 std(dtrd(BN))	1.6412	8.8103
2	0.4 std(dtrd(BN))	1.6345	20.101
3	0.4 std(dtrd(BN))	1.6013	NaN
1	0.3 std(dtrd(BN))	1.9272	8.9028
2	0.3 std(dtrd(BN))	1.9115	20.101
3	0.3 std(dtrd(BN))	1.8220	NaN
1	0.2 std(dtrd(BN))	2.3290	9.6780
2	0.2 std(dtrd(BN))	2.2828	20.101
3	0.2 std(dtrd(BN))	1.9870	NaN
1	0.02 std(dtrd(BN))	4.3411	20.101
2	0.02 std(dtrd(BN))	1.3888	NaN
3	0.02 std(dtrd(BN))	0.0321	NaN
1	0.002 std(dtrd(BN))	3.4545	20.101
2	0.002 std(dtrd(BN))	0.0292	NaN
3	0.002 std(dtrd(BN))	0.00001	NaN
